# Pyoderma gangrenosum: a review with special emphasis on Latin America literature^☆^^☆☆^

**DOI:** 10.1016/j.abd.2019.06.001

**Published:** 2019-11-01

**Authors:** Milton José Max Rodríguez-Zúñiga, Michael S. Heath, João Renato Vianna Gontijo, Alex G. Ortega-Loayza

**Affiliations:** a Instituto de Evaluación de Tecnologías Sanitarias, Lima, Peru; b Oregon Health and Sciences University, Portland, United States; c Adult Health Postgraduate Program, School of Medicine, Universidade Federal de Minas Gerais, Belo Horizonte, MG, Brazil; d Department of Dermatology, Oregon Health and Sciences University, Portland, OR, United States

**Keywords:** Inflammatory bowel diseases, Latin America, Pyoderma gangrenosum, Skin ulcer

## Abstract

Pyoderma gangrenosum is a neutrophilic dermatosis characterized by chronic ulcers due to an abnormal immune response. Despite the existence of diagnostic criteria, there is no gold standard for diagnosis or treatment. In Latin America, recognizing and treating pyoderma gangrenosum is even more challenging since skin and soft tissue bacterial and non-bacterial infections are common mimickers. Therefore, this review aims to characterize reported cases of pyoderma gangrenosum in this region in order to assist in the assessment and management of this condition. Brazil, Mexico, Argentina, and Chile are the countries in Latin America that have reported the largest cohort of patients with this disease. The most frequent clinical presentation is the ulcerative form and the most frequently associated conditions are inflammatory bowel diseases, inflammatory arthropaties, and hematologic malignancies. The most common treatment modalities include systemic corticosteroids and cyclosporine. Other reported treatments are methotrexate, dapsone, and cyclophosphamide. Finally, the use of biological therapy is still limited in this region.

## Introduction

Pyoderma gangrenosum (PG) is an inflammatory disease, most commonly characterized by painful cutaneous ulcers with irregular, violaceous borders located on the lower limbs. It is frequently associated with inflammatory bowel diseases (IBD), inflammatory arthropathies, and hematologic malignancies.^1^, ^2^ The worldwide incidence is estimated to be around two to three cases per 100,000 habitants per year,^3^ but this might be underestimated due to lack of a diagnostic gold standard. Pathogenesis is not well understood, but studies have suggested an abnormal immune response in patients with genetic predisposition, hence PG is classified within the spectrum of neutrophilic and auto-inflammatory syndromes.^4^, ^5^

Other pathologic conditions in the clinical differential diagnosis – including infections, vasculitis/vasculopathy, and neoplastic disorders – should be ruled out with the assistance of laboratory testing, as well as histopathologic and microbiological studies.^6^ First-line treatment includes systemic corticosteroids and cyclosporine. Second-line and third-line therapeutic options comprise immunosuppressive, immunomodulatory, and biologic agents.^7^ The present study aimed to review the literature in order to recommend the best approach when facing patients with PG in Latin America (LA).

## Methods

A systematic review was performed of the case-reports and case-series studies of PG from countries in LA published in MEDLINE (PubMed) and LILACS from inception to October 2018. The search strategies are available in Table 1.

**Table 1:** Search strategy

Database	PubMed: (https://www.ncbi.nlm.nih.gov/pubmed/) Date: October 23rd, 2018.		Results
Strategy	#1	Search (chile OR brazil OR peru OR brasil OR colombia OR mexico OR ecuador OR venezuela OR uruguay OR cuba OR puerto rico OR costa rica OR latin america OR argentina)	702779
	#2	Search Pyoderma Gangrenosum[tiab]	2853
	#3	Search (#1 AND #2)	61

Database	LILACS (http://pesquisa.bvsalud.org/portal/) Date: October 23rd, 2018.		Results
Strategy	#1	(tw:(pyoderma gangrenosum)) AND (instance:“regional”) AND (db:(“LILACS”))	141

### Epidemiology

PG is considered a rare disease of with estimated prevalence of two to three cases per 100,000 people and an adjusted incidence rate of 0.63 per 100,000 person-years. Risk of death is three times higher than general controls.^8^ It tends to have a slight predominance for females.^8^, ^9^ Differences in comorbid conditions and differential diagnoses to consider vary significantly depending on geographic regions and local disease prevalence. Reports from LA are scarce and mostly consist of case reports or case series.^10^

### Pathogenesis

Neutrophilic dysfunction has been implicated in the pathogenesis of PG.^9^ In addition, pathergy, which is defined as a nonspecific increase of neutrophil activity reaction, is present in other neutrophilic dermatoses (*e.g.*, Behcet's disease and Sweet's syndrome) but it has been described in at least 30% of patients.^11^, ^12^ Neutrophilic dysfunction shares the same pro-inflammatory effectors found in auto-inflammatory syndromes. Both are characterized by an over-activated innate immune system leading to the increased assembly of inflammasomes.^2^ Inflammasomes are responsible for the activation of the caspase 1, a protease that cleaves the pro-interleukin IL-1β into functionally active IL-1β. The overproduction of IL-1β triggers the release of several pro-inflammatory cytokines and chemokines, inducing the recruitment and activation of neutrophils and subsequent neutrophil-mediated inflammation.^5^ IL-17 appears to be crucial in the recruitment of neutrophils in auto-inflammation and acts synergistically with tumor necrosis factor (TNF).^13^, ^14^ Finally, IL-1β, IL-17, and TNF-α activate and increase the production of matrix metalloproteinases (MMPs), which are overexpressed in the inflammatory infiltrate of PG, causing an inflammatory insult and the consequent destruction of the involved tissue.^2^, ^5^

In conclusion, PG is the result of innate immune system over-activation *via* inflammasomes, coupled with the activation of the adaptive immune system, triggered by an external insult (*e.g.*, pathergy) and/or a possible internal trigger in a genetically predisposed individual.^15^

### Histopathologic findings

PG has non-specific histopathologic findings. Presence of perifollicular inflammation, edema, and neutrophilic inflammation are the initial features seen in untreated and expanding PG lesions. Polymorphonuclear leukocyte infiltration can lead to abscess formation and necrosis of the tissue with mixed inflammatory infiltrate. Additional findings may include giant cells, secondary thrombosis of small- and medium-sized vessels, and hemorrhage. Secondary leukocytoclastic vasculitis is present in around 40% of cases. Direct immunofluorescence also yields non-specific findings such as deposition of IgM, C3, and fibrin in the papillary and reticular dermal vessels. Due to the non-specific findings, skin biopsies are more useful to rule out other causes of ulceration that may present with similar clinical findings, such as infections, vasculitis, vasculopathies, or malignancies.^12^, ^16^, ^17^, ^18^

### Clinical features

**Figure 1:**
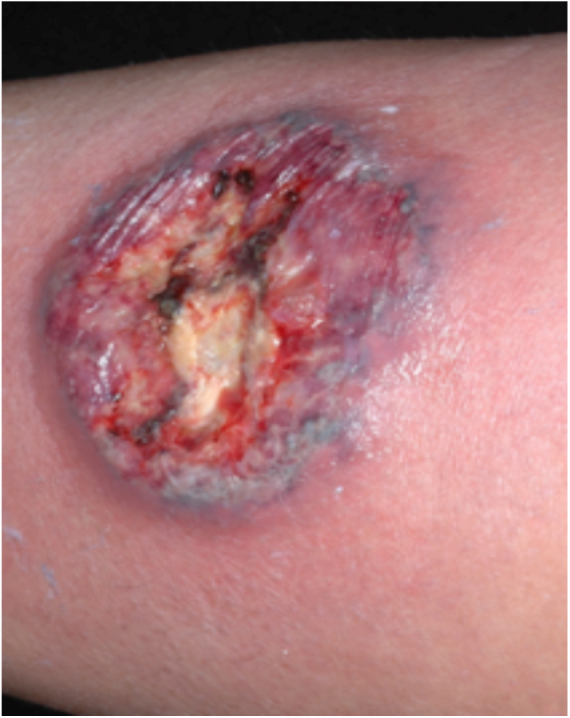
Classic or ulcerative pyoderma gangrenosum.

**Figure 2:**
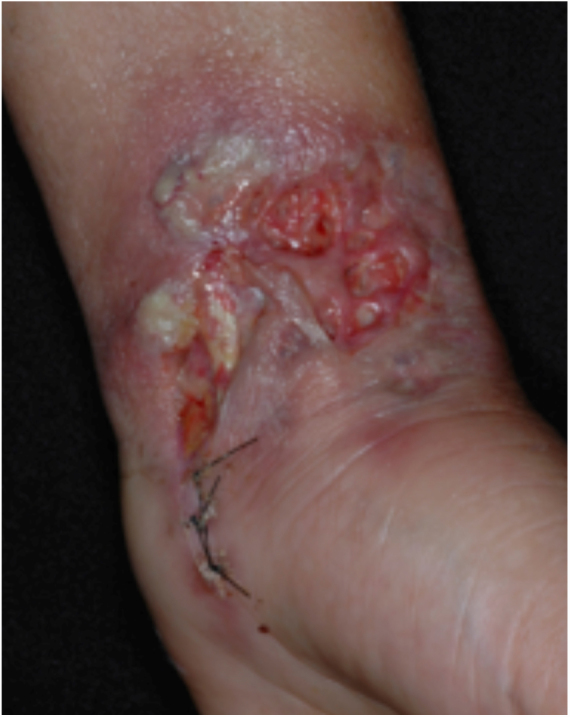
Classic or ulcerative pyoderma gangrenosum.

**Figure 3:**
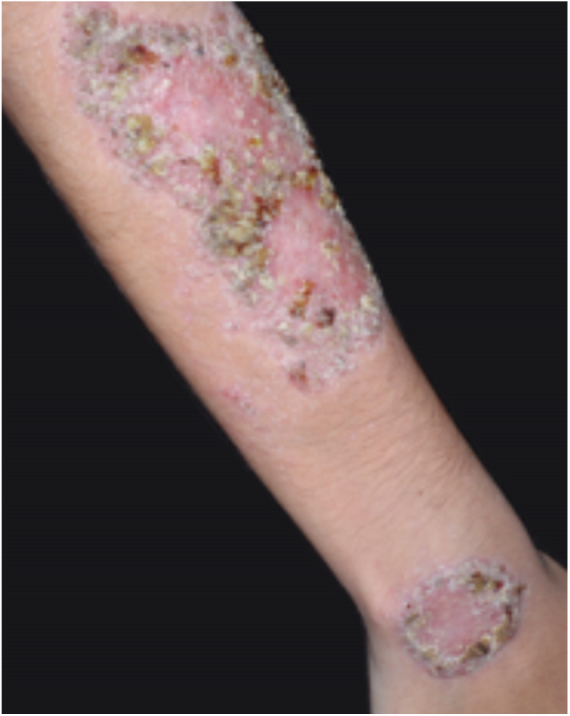
Vegetative or verrucous pyoderma gangrenosum in a patient with Behcet's disease.

**Figure 4:**
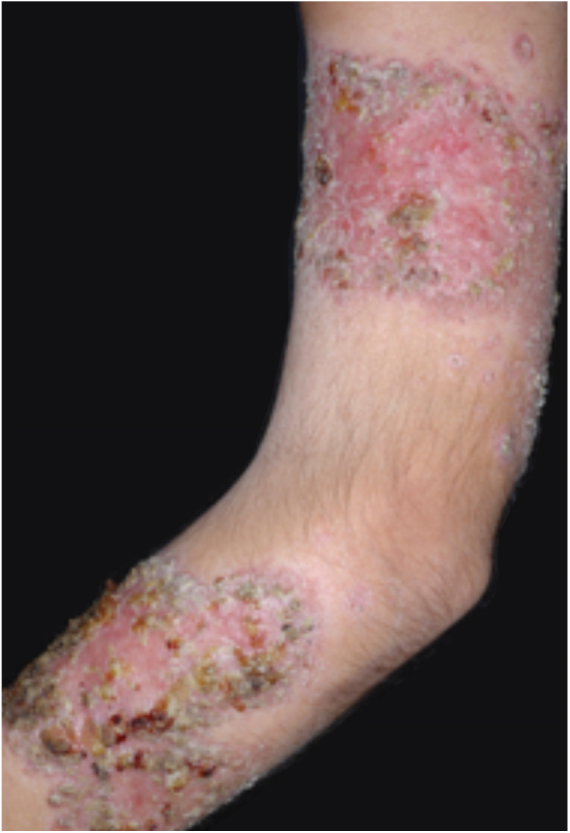
Vegetative or verrucous pyoderma gangrenosum in a patient with Behcet's disease.

**Figure 5:**
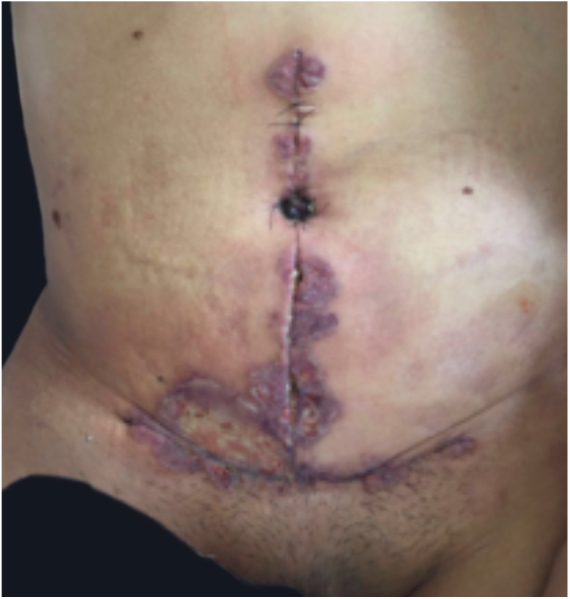
Pyoderma gangrenosum after abdominoplasty.

**Figure 6:**
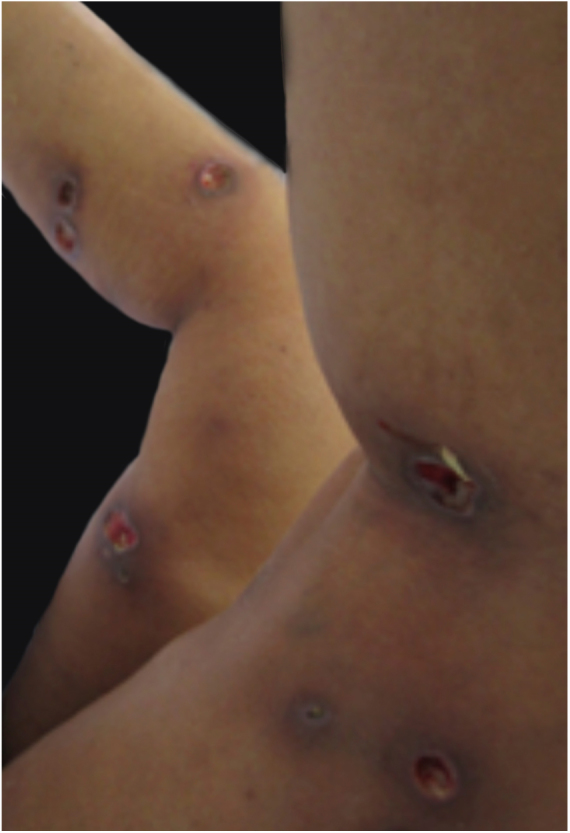
Multiple pyoderma gangrenosum ulcers at the sites of sclerotherapy injections.

PG is classified into four clinical subtypes: classic (ulcerative) (Figure 1, Figure 2), bullous, pustular, and vegetative (Figure 3, Figure 4). Ulcerative or classic PG often starts as an inflammatory erythematous violaceous pustule of a few millimeters in size, which enlarges forming an ulceration that gradually increases both in size and depth. The ulcer discharges a purulent and hemorrhagic exudate, easily detectable by applying pressure on its border. The purulent and malodorous exudate can be attributable to a bacterial colonization or to an actual superinfection. The border is well demarcated, elevated, and slowly progressive, with a violaceous color. An erythematous, edematous, and infiltrated halo extends up to 2cm from the border of the ulcer.^2^, ^5^, ^19^ The lesion is usually solitary, but multiple ulcers can occur; they are typically painful, ranging from a few millimeters to 30cm or more, localized mostly in extensor surface of the legs, but they can affect any anatomic site. They may be deep enough to expose tendons, fasciae, and muscles.^5^, ^20^ Lesions start in healthy skin and may be provoked by trauma (pathergy). Therefore, postoperative PG (Figure 5, Figure 6), peristomal PG, and worsening of lesions after sharp or surgical debridement frequently occur.^21^ The ulcer can propagate rapidly, showing a serpentine configuration.^3^, ^19^ PG has been classically associated with inflammatory colitis (IBD and diverticulitis), hematological malignancies (myelodysplastic syndrome, monoclonal gammopathy, chronic myeloid leukemia, *etc.*), autoimmune inflammatory disease (seronegative polyarthritis, rheumatoid arthritis), and solid tumors (prostate and colon adenocarcinoma).^4^, ^8^ Finally, sterile neutrophilic infiltrates have been found to affect internal organs supporting the concept of PG being a systemic disease.^22^

## Results

In LA, 118 studies were found from 1981 to 2018, with 232 cases of PG. Brazil was the country with the largest report of case-series, with 96 (41.4%) cases of PG. The next highest total was from Argentina, which has 69 (29.7%) reported cases of PG, followed by Chile and Mexico, which have a similar number of reported cases, with 21 (9.1%) and 17 (7.3%), respectively. In addition, the results of the systematic review show that ulcerative PG was the most frequent type of PG reported, and that the others had similar prevalence. Bullous, vegetative (granulomatous), and pustular PG were reported in nine (3.9%), eight (3.5%), and five (2.1%) cases. The rest of PG cases did not report the subtype (Table 2).

**Table 2:** Prevalence of Latin American pyoderma gangrenosum cases reported in the literature

	Total (*n*)	%	Ulcerative	Granulomatous	Pustular	Bullous
Brazil	96	41.38	76	3	1	3
Argentina	69	29.74	57	2	2	3
Bolivia	1	0.43	1	‒	‒	‒
Chile	21	9.05	20	‒	1	‒
Colombia	16	6.90	14	‒	‒	‒
Costa Rica	1	0.43	1	‒	‒	‒
Cuba	2	0.86	1	1	‒	‒
Mexico	17	7.33	5	1	‒	‒
Paraguay	1	0.43	‒	‒	‒	1
Peru	5	2.16	2	‒	1	2
Venezuela	3	1.29	2	1	‒	‒
TOTAL	232	100	179	8	5	9
%	100	‒	77.16	3.45	2.16	3.88

In addition, the systematic review found that there was a high prevalence of PG cases associated with comorbidities. Overall, 149 (64.5%) and 37 (16%) of the patients included in the analysis had PG associated to a condition or surgery, respectively. In the rest of PG patients, no other conditions or disease associations were reported. IBD was the most frequent conditions associated (53/149, 35.6%), then ulcerative colitis (UC) (32/149, 21.5%) and Crohn's disease (10/149, 6.7%). Other inflammatory diseases were also frequent (54/149, 36.2%); among them, rheumatoid arthritis (RA) (17/149, 11.4%), antiphospholipid syndrome (APS) (15/149, 10.1%), systemic erythematous lupus (4/149, 2.7%), and Takayasu's arteritis (4/149, 2.7%) were reported. Several malignances were reported in association with PG (19/149, 12.8%), in particular hematologic malignancies (14/149, 9.4%) and solid-organ malignancies (5/149, 3.4%). Other conditions were also reported (23/149, 15.4%), including the presence of pulmonary nodules (5/149, 3.4%) of unknown etiology. In regard to surgical procedures, reduction mammoplasty (9/149, 6.0%), laparotomy (6/149, 4.0%), and skin grafting (4/149, 2.7%) were the more frequent triggers of PG. It is important to mention that due to the limited information in the reports and the scarce number of PG cases, only descriptive statistical analysis was performed (Table 3).

**Table 3:** Prevalence of the conditions associated with pyoderma gangrenosum reported in the Latin American literature

Condition	*n*	%
*IBD*		
No specified	11	7.4
UC	32	21.5
CD	10	6.7
Subtotal	53	35.6

*Malignancy*		
Hematologic malignancy	14	9.4
Solid-organ malignancy	5	3.4
Subtotal	19	12.8

*Inflammatory*		
Antiphospholipid syndrome	15	10.1
Rheumatoid arthritis	17	11.4
Lupus erythematosus	4	2.7
Takayasu's arteritis	4	2.7
Other	14	9.4
Subtotal	54	36.2

*Other conditions*		
Pulmonary nodules	5	3.4
Cocaine consumption	2	1.3
Diverticular disease	2	1.3
Pregnancy	2	1.3
Other	12	8.1
Subtotal	23	15.4
Total	149	64.5^a^

*Secondary to surgery*		
Reduction mammoplasty	9	22.5
Laparotomy	6	15.0
Abdominoplasty	4	10.0
Skin grafts	4	10.0
Total surgery	37	16.0^b^

a Total of patients with comorbidities/total of patients with PG × 100.

b Total of patients with PG secondary to surgery/total of patients with PG × 100. IBD, inflammatory bowel disease; UC, ulcerative colitis; CD, Crohn's disease.

Thus, the systematic review of PG case-series from LA helped to elucidate the main associated conditions. In Table 4, all the studies where PG was reported to be associated with clinical or surgical conditions are listed.

**Table 4:** Conditions associated with pyoderma gangrenosum (PG) reported in the Latin American literature

*n*	Author	Year	Country	Number of patients	Clinical type	Associated disease	Secondary to surgery or drugs
1	Corti et al.^59^	1981	Argentina	1	Ulcerative	Crohn's disease	No
2	Della-Giovanna et al.^60^	1991	Brazil	4	Ulcerative	Two patients with ulcerative colitis, one with pregnancy	No
3	Moreno et al.^61^	1994	Argentina	2	Ulcerative	Two patients with rheumatoid arthritis	No
4	Vignale et al.^62^	1996	Argentina	4	Ulcerative	Three patients with Crohn's disease, two with seronegative arthritis, and one with ulcerative colitis	No
5	Saraceno et al.^63^	2002	Argentina	6	Ulcerative	One patient with myelodysplastic syndrome, one with rheumatoid arthritis, one with hemophagocytic syndrome and hepatitis C	No
6	Plaza^64^	2004	Argentina	1	Ulcerative	Rheumatoid arthritis	No
7	Simon et al.^65^	2005	Argentina	1	Ulcerative	Ulcerative colitis	Peristomal after total colectomy
8	Vazquez et al.^66^	2009	Argentina	1	Ulcerative	Chronic osteomyelitis	Knee prosthesis replacement
9	Sommerfleck et al.^67^	2014	Argentina	1	Not reported	Ulcerative colitis	No
10	Achenbach et al.^68^	2014	Argentina	1	Ulcerative	Crohn's disease	Sulfasalazine
11	Curmona et al.^69^	2014	Argentina	2	Ulcerative	One patient with metastatic ovarian cancer	Abdominal tumor resection
12	Pereyra et al.^70^	2014	Argentina	1	Ulcerative	Renal transplant	No
13	Fassi et al.^71^	2015	Argentina	1	Ulcerative	Rheumatoid arthritis and ulcerative colitis	No
14	Silva-Feistner et al.^72^	2015	Argentina	1	Ulcerative	None	Cesarean
15	Galimberti et al.^73^	2016	Argentina	1	Ulcerative	Ulcerative colitis	No
16	Vacas et al.^1^	2017	Argentina	27	Ulcerative	Ten patients with IBD, seven with hematologic malignances, five with rheumatoid or seronegative arthritis, and one with cocaine consumption	No
				2	Bullous		
				2	Pustular		
17	Vega et al.^74^	2018	Argentina	1	Ulcerative	Retroperitoneal fibrosis	Exploratory mid-laparotomy
18	Vacas et al.^75^	2018	Argentina	1	Bullous	Acute and chronic myeloid leukemia	No
19	Pires et al.^76^	1987	Brazil	1	Ulcerative	Chronic hepatitis	No
20	Lana et al.^77^	1990	Brazil	1	Ulcerative	Multiple sclerosis	No
21	Pessato et al.^78^	1996	Brazil	9	Not reported	Five patients with comorbidities	No
22	Souza et al.^79^	1999	Brazil	11	Predominantly ulcerative	One patient with ulcerative colitis, one with rheumatoid arthritis, one with Grave's disease, one with diabetes mellitus	No
23	Cabral et al.^80^	2004	Brazil	4	Ulcerative	Four patients with ulcerative colitis, one with additional primary sclerosing cholangitis, one with seronegative arthritis	No
24	Martinez et al.^81^	2005	Brazil	1	Ulcerative	Ulcerative colitis	No
25	Costa et al.^82^	2005	Brazil	1	Ulcerative	Rheumatoid Arthritis	No
26	Fraga et al.^83^	2006	Brazil	1	Superficial granulomatous	Psoriasis	No
27	Coltro et al.^84^	2006	Brazil	1	Ulcerative in donor site	None	Skin graft for varicose ulcers
28	Batista et al.^85^	2006	Brazil	1	Bullous	Myelodysplastic syndrome	No
29	Meyer et al.^86^	2006	Brazil	1	Ulcerative	Crohn's disease	Infraumbilical median laparotomy
30	Franca et al.^87^	2006	Brazil	1	Ulcerative	Gastric adenocarcinoma	No
31	Burkieviecz et al.^88^	2007	Brazil	1	Ulcerative	Systemic lupus erythematosus/antiphospholipid syndrome	No
32	Barbato et al.^89^	2008	Brazil	13	Ulcerative	Two patients with Crohn's disease, two with diabetes, two with collagenosis, one with leukemia.	No
				2	Superficial granulomatous		
				1	Bullous		
33	Tinoco et al.^90^	2008	Brazil	1	Ulcerative	Acne	Isotretinoin
34	Dornelas et al.^91^	2008	Brazil	1	Ulcerative	None	Reduction mammoplasty and abdominoplasty
35	Bonamigo et al.^92^	2008	Brazil	1	Ulcerative	None	Breast implant with silicone prosthesis and facial surgery
36	Coelho et al.^93^	2009	Brazil	2	Ulcerative	Inflammatory arthritis/splenomegaly	No
37	Berbert et al.^94^	2009	Brazil	1	Superficial granulomatous	None	Donor site after skin grafting for burns.
38	Furtado et al.^95^	2010	Brazil	1	Ulcerative	None	Reduction mammoplasty and abdominoplasty
39	Avelar et al.^96^	2011	Brazil	1	Ulcerative	Breast cancer	Breast quadrantectomy and radiotherapy
40	Fonseca et al.^97^	2011	Brazil	1	Bullous	Ulcerative colitis	No
41	Grillo et al.^98^	2012	Brazil	1	Ulcerative	None	Reduction mammoplasty
42	Maia et al.^99^	2012	Brazil	1	Ulcerative	Cocaine use	No
43	Cunha et al.^100^	2012	Brazil	1	Ulcerative	None	Laparoscopy
44	Bittencourt et al.^101^	2012	Brazil	1	Ulcerative	Pulmonary nodules	No
45	Andrade et al.^102^	2012	Brazil	1	Ulcerative	Inflammatory bowel disease	No
46	Carvalho et al.^103^	2013	Brazil	1	Ulcerative	Splenic and renal impairment	No
47	Kruger et al.^104^	2013	Brazil	1	Ulcerative	Ulcerative colitis	No
48	Soares et al.^105^	2013	Brazil	1	Ulcerative	None	Reduction mammoplasty and abdominoplasty
49	Beber et al.^106^	2014	Brazil	1	Ulcerative	Rheumatoid arthritis	No
50	Marchiori et al.^107^	2014	Brazil	1	Ulcerative	Pulmonary nodules	No
51	Rosseto et al.^108^	2015	Brazil	1	Ulcerative	None	Abdominoplasty
52	de Souza et al.^109^	2015	Brazil	1	Ulcerative	Myelodysplastic syndrome	No
53	Sempertegui et al.^110^	2015	Brazil	1	Ulcerative	Breast cancer	Breast quadrantectomy and radiotherapy
54	Soncini et al.^111^	2016	Brazil	1	Ulcerative	None	Augmentation mastopexy
55	Dantas et al.^55^	2017	Brazil	1	Ulcerative	Autoimmune hepatitis	No
56	Freitas et al.^112^	2017	Brazil	1	Ulcerative	Iliac vein compression syndrome	No
57	Gabe et al.^113^	2018	Brazil	1	Ulcerative	Pyogenic arthritis	No
58	Bittencourt et al.^114^	2018	Brazil	1	Ulcerative	Multiple myeloma	Autologous stem cell transplantation and then laparoscopic cholecystectomy
59	Clemente et al.^115^	2018	Brazil	2	Ulcerative	Two patients with atypical Takayasu's arteritis	No
60	Perez et al.^116^	2001	Chile	2	Ulcerative	None	Cesarean
61	Lopez de Maturana et al.^54^	2001	Chile	3	Ulcerative	Two patients with diverticular disease, one patient with Crohn's disease, one patient with rheumatoid arthritis	No
				1	Pustular		
62	Hevia et al.^117^	2004	Chile	1	Ulcerative	Ulcerative colitis	Peristomal after total colectomy
63	Eulufi et al.^118^	2006	Chile	3	Ulcerative	None	Two patients after skin graft, one patient after reduction mammoplasty
64	Calderon et al.^119^	2011	Chile	1	Ulcerative	Ulcerative colitis	No
65	Fernandez-Castillo et al.^120^	2012	Chile	1	Ulcerative	Wegener granulomatosis/chronic kidney disease	Arteriovenous fistula
66	Gosch et al.^121^	2012	Chile	1	Ulcerative	None	Reduction mammoplasty
67	Melo and Fernandez ^122^	2013	Chile	1	Ulcerative	None	Foot and ankle surgery
68	Calderon et al.^123^	2013	Chile	3	Ulcerative	None	After reduction mammoplasty
69	Bannura et al.^124^	2014	Chile	1	Ulcerative	Ulcerative colitis	Laparotomy/ileostomy
70	Erlij et al.^125^	2018	Chile	1	Ulcerative	Erythema induratum and large B-cell non-Hodgkin's lymphoma	No
71	Penaloza et al.^126^	1988	Colombia	1	Ulcerative	Ulcerative colitis	No
72	Restrepo et al.^127^	2006	Colombia	2	Ulcerative	Two patients with ulcerative colitis	No
73	Jaime-Lopez et al.^128^	2009	Colombia	1	Ulcerative	Ulcerative colitis	Infliximab
74	Cañas et al.^129^	2010	Colombia	7	Ulcerative	Seven patients with antiphospholipid syndrome	No
75	Cadavid et al.^130^	2012	Colombia	1	Ulcerative	Ulcerative colitis	None
76	Severiche et al.^131^	2014	Colombia	1	Ulcerative	Breast phyllodes tumor (paraneoplastic)	No
77	Acon-Ramirez et al.^132^	2017	Costa Rica	1	Ulcerative	Rheumatoid arthritis	No
78	Moreira-Preciado et al.^133^	2001	Cuba	1	Superficial granulomatous	Renal failure	No
79	Hernandez-Urra et al.^134^	2010	Cuba	1	Ulcerative	Ulcerative colitis	No
80	Zonana-Nacach et al.^135^	1994	Mexico	2	Not reported	Two patients with rheumatoid arthritis	No
81	Reynoso-von Dratein et al.^58^	1997	Mexico	9	Not reported	Three patients with rheumatoid arthritis and two patients with systemic lupus erythematosus	No
82	Chacek et al.^136^	1998	Mexico	1	Ulcerative	Inferior cava vein syndrome due to thrombosis and antiphospholipid syndrome	No
83	Muñiz-Gonzalez et al.^137^	2007	Mexico	1	Ulcerative	Ulcerative colitis, diverticular disease	No
84	Contreras-Ruiz et al.^138^	2008	Mexico	1	Ulcerative	Rheumatoid arthritis	No
85	Barrera-Vargas et al.^139^	2015	Mexico	2	Ulcerative	Two patients with pulmonary nodules and Takayasu's arteritis	No
86	Contreras-Verduzco et al.^140^	2018	Mexico	1	Superficial granulomatous	Pulmonary nodules and nodular scleritis	No
87	Real-Delor et al.^141^	2011	Paraguay	1	Bullous	Ulcerative colitis	No
88	Dominguez et al.^142^	2009	Peru	1	Pustular	Ulcerative colitis	No
89	Carrillo-Nanez et al.^143^	2014	Peru	1	Bullous	Ulcerative colitis	No
90	Deza-Araujo et al.^144^	2014	Peru	1	Ulcerative	Systemic lupus erythematosus	No
91	Fermin et al.^145^	1989	Venezuela	2	Ulcerative	One patient with ulcerative colitis	No
92	Valecillos et al.^146^	1998	Venezuela	1	Superficial granulomatous	Pregnancy	No

### Infectious PG-mimickers to be considered in LA and/or from LA

**Figure 7:**
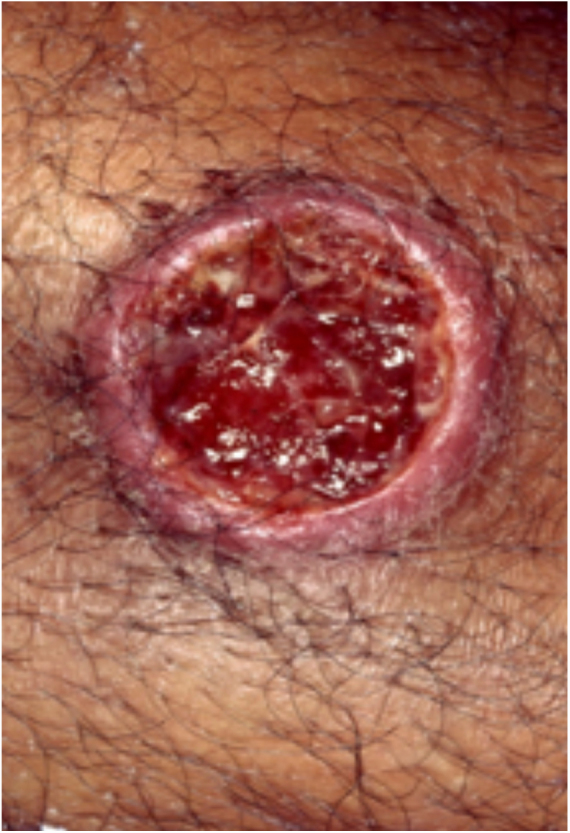
Cutaneous leishmaniasis lesions can mimic classic pyoderma gangrenosum lesions.

**Figure 8:**
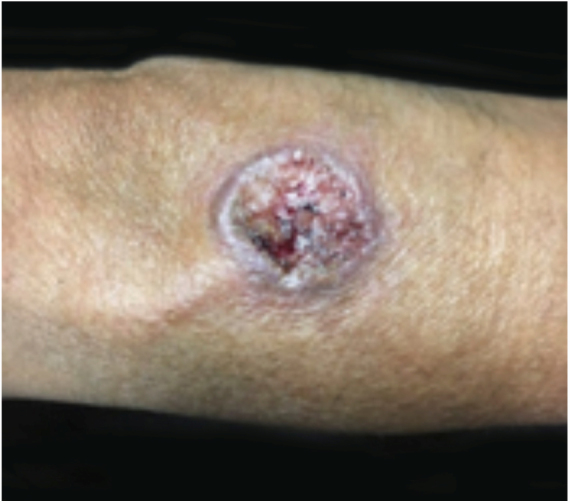
Cutaneous leishmaniasis lesions can mimic classic pyoderma gangrenosum lesions.

In general, ulcerative entities resembling PG fall into one of six disease categories: (a) primary deep cutaneous infections (*e.g.*, sporotrichosis, cutaneous tuberculosis, leishmaniasis, *etc.*) (Figure 7, Figure 8); (b) vascular occlusive or venous disease (*e.g.*, APS, venous stasis ulceration, *etc*.); (c) vasculitis (*e.g.*, Wegener's granulomatosis, polyarteritis nodosa, *etc.*); (d) malignant processes involving the skin (*e.g*., angiocentric T-cell lymphoma, anaplastic large-cell T-cell lymphoma, *etc*.); (e) drug-induced or exogenous tissue injury (factitial disorder, loxoscelism, *etc.*); and (f) other inflammatory disorders (cutaneous Crohn's disease, ulcerative necrobiosis lipoidica, *etc.*). However, in LA, the most common differential diagnoses include deep cutaneous infections, vascular occlusive disease, and metabolic disorders.^23^ It is crucial for the management of PG in this region to rule out infection, as immunosuppressive medications used to treat PG may be contraindicated in these patients.^24^ Depending on the specific region, infectious ulcers can be caused by *Leishmania* parasites, atypical *Mycobacterium* species, deep fungal infections (sporotrichosis, chromoblastomycosis and, mycetoma), myasis, and cutaneous amebiasis.^3^

Cutaneous leishmaniasis (CL) is a common skin disease in developing countries, such as Brazil and Peru.^25^ Lesions may start as a papule or a nodule that develops into an ulcer with or without a scar (Figure 7, Figure 8). They are usually painless, but when painful, secondary infection is generally present. The initial diagnosis of CL is based on the clinical presentation and the patient's history of visiting an endemic area. Diagnostic work up includes multiple components in order to provide the highest likelihood of confirmation. Biopsy specimens should be obtained for impression smears, histopathologic slides with hematoxylin and eosin, Giemsa stain and special stains for other microbes, culture, and polymerase chain reaction (PCR) analysis if diagnosis is challenging.^26^

Ulcerating cutaneous lesions can be caused by mycobacterial infections including *Mycobacterium ulcerans*, *M. marinum*, and *M. tuberculosis. M. ulcerans* is the third most common agent of mycobacterial disease (after tuberculosis and leprosy) and the most common mycobacterium causing cutaneous ulceration.^27^ Buruli ulcers, which are caused by *M. ulcerans*, are endemic in foci in West Africa and have been reported as an imported disease in countries of LA such as Peru, Brazil, Guiana, and Mexico.^28^ Microscopy and PCR are used for routine diagnosis, while culture is less useful given time requirements and lack of species specificity.^29^ Treatment is generally surgical, although a combination of rifampicin and streptomycin may be effective in the early stage.

Cutaneous tuberculosis (CTB) is an infection caused by *M. tuberculosis* complex, *M. bovis*, and in immunocompromised hosts, by bacillus Calmette-Guérin (BCG) vaccination.^30^ An increase in its incidence has been described in several countries of LA in recent years, especially in urban centers and regions with high prevalence of human immunodeficiency virus (HIV) infection. Lupus vulgaris is a form of CTB that initiates as smoldering papular or tuberous lesions and progresses to plaques with necrosis and ulceration, with or without cicatricial deformities and mutilations.^31^ Diagnostic approaches to CTB include skin biopsy with acid fast bacilli stain and culture, as well as PCR amplification testing of biopsy tissue.^32^

Sporotrichosis is one of the most common deep mycoses; it may be accompanied by ulceration.^33^ Most cases of sporotrichosis are currently being reported in South and Central America, with recent outbreaks transmitted by cats.^34^ The primary cutaneous lesions may appear as papular, nodular, or pustular lesions that develop into either a superficial ulcer or a verrucous plaque. During progression, the lymphocutaneous form displays multiple subcutaneous nodules that are formed along the course of locally draining lymphatics (sporotrichoid spread). In contrast, the localized form shows no lymphatic spread and is characterized by indurated or verrucous plaques and occasional ulcers.^35^, ^36^ Diagnosis of sporotrichosis is primarily clinical due to its distinguished presentation, but in difficult cases, culture is the gold standard.^37^Sporotrichosis is treated with systemic itraconazole.^38^

Mycetoma is a chronic subcutaneous fungal infection caused by inoculation *via* organic matter such as splinters or thorns. Given the ubiquitous nature of causative fungi, there may be a genetic predisposition to developing the disease.^39^ In South America, the most common pathogens are *Trematosphaeria grisea*, *Madurella mycetomatis*, and *Scedosporium apiospermum*. The clinical manifestations of mycetoma include painless slow growing subcutaneous nodules, which may evolve into necrotic abscesses with sinus tracts. Lesions are most commonly located on exposed areas, especially the lower extremities. The expression of black granules and debris from the lesions is highly suggestive of fungal mycetoma.^40^ Diagnostic work up includes tissue microscopy using potassium hydroxide, as well as deep tissue biopsies for culture on Sabouraud dextrose agar kept at both room temperature and 37 °C. As fungal cultures can take several weeks, histopathologic evaluation may expedite the diagnosis but may not aid in identifying the causative species.

Furuncular myiasis is most commonly caused by larval tissue penetration by *Dermatobia hominis*, the botfly, and *Cordylobia anthropophaga*, the tumbu fly. Lesions slowly evolve into periodically painful nodules. They may present with multiple nodules containing larvae, and removal of the larvae typically relieves symptoms.

Cutaneous amebiasis is a rare extracutaneous manifestation of *Entamoeba histolytica* infection. It can occur independently through direct inoculation or along with other tissue involvement. There is predilection of the disease in the perineal region due to direct inoculation from stool, leading to ulcerations, but lesions can be present anywhere on the body.^41^ Notable clinical features include one or more painful and malodorous ulcers with a necrotic base.^42^ The edges of the lesions are often raised and red in color. Progression of the ulcers is often rapid, with destruction in all planes.^42^ Confirmation of the diagnosis can be made with identification of the pathogen on tissue biopsy of the ulcer. Cytologic smears may also be useful in identifying the trophozoites.^42^

Chromoblastomycosis infection is causes by fungal spore implantation into the skin at sites of trauma. The fungus is ubiquitous in the soil and vegetation and therefore is highly associated with trauma occurring in the outdoors.^43^, ^44^ This inoculation leads to a chronic granulomatous reaction. The infection initially begins as an inflamed macule, which evolves into a papule and eventually develops into one of several morphologic subtypes, including nodular, verrucous, tumorous, cicatricial, or plaque types.^45^ Disease progression is very slow and is usually limited to the skin and subcutaneous tissue. Due to the mechanism of inoculation, it most commonly occurs on the lower extremities. One rare complication in chronic lesions is squamous cell carcinoma transformation.^44^, ^45^ Diagnosis is made by identifying muriform cells on microscopy.^44^, ^45^, ^46^, ^47^, ^48^ Samples can be obtained *via* scrapings, tape preparation, wet mounts, tissue biopsy, or culture. Treatment is dependent on the stage at diagnosis; early lesions can be treated effectively with surgery or cryotherapy. Pharmacologic treatment options are systemic antifungals for refractory or extensive disease.^44^, ^47^, ^49^

### Treatment of PG in LA

There is no gold standard therapy for PG, but treatment should be guided by extension and depth of the ulcer, associated systemic diseases, the patient's performance status, and availability of medications.^3^, ^50^ Topical therapy is the first choice for small lesions in the early stages (papules, pustules, nodules, or superficial ulcers). It includes dressings, topical immunomodulators, and intralesional corticosteroid injections.^51^ A report from Chile showed an excellent and rapid respond to cyclosporin 5%, clobetasol 0.05%, and gentamycin 0.2% ointment applied twice daily, after 18 weeks.^52^

In patients with severe forms of PG, or with rapid expansion and resistance to topical treatment, systemic therapy is the treatment of choice. Corticosteroids are the first line drugs in the acute phase and should be initiated at high doses (1–2 mg/kg).^3^, ^53^ A steroid-sparing drug should be used in the initial phases to minimize long-term steroid toxicity and side effects. Agents including dapsone, sulfasalazine, methotrexate, azathioprine, mycophenolate mofetil, cyclophosphamide, cyclosporine, and biologics may be employed.^2^, ^53^

In LA, systemic corticosteroids are, by far, the most frequent first-line therapy. In a cohort of patients with PG, systemic corticosteroids were administered to 87% of patients (*n* = 27), at a dose-range of 1–1.5 mg/kg/day, for two to 14 months.^1^ In another cohort, these drugs were initiated in 81.8% of the patients (*n* = 9); after 60 months, only two patients had been recurrence free, while the others had multiple recurrences, treated with sulfamethoxazole and trimethoprim, minocycline, topical corticosteroids, and cyclophosphamide.^54^ In addition, corticosteroids and local treatment were preferred when PG was associated with autoimmune diseases, such as autoimmune hepatitis.^55^

Cyclosporine is less frequently used as a first-line therapy due to its side effects. Cyclosporine monotherapy induces a rapid remission of the disease at a dose of 3–5 mg/kg/day after a few weeks of treatment and complete resolution of the lesion after one to three months, but long-term therapy might be problematic.^3^ In one report from LA, it was used in 13% (*n* = 4) of the patients, for one to six months, with a dose of 1.5–3 mg/kg/day, with no relapses after four years of treatment.^1^

Experience with methotrexate in LA is scarce, and it is generally used as a steroid-sparing drug in severe or refractory disease.^56^ However, a case report from Chile showed a remarkable response with intralesional methotrexate. After 40 days of oral prednisolone, followed by eight weeks of 10 mg weekly intramuscular methotrexate, the PG ulcers failed to improve. After seven injections of methotrexate (25 mg/week) administered intralesionally in the erythematous border of the ulcers, almost 90% of the ulcer was healed.^57^

In Mexico, an open-label trial assessed intravenous bolus cyclophosphamide in a dose of 500 mg/m^2^ of body surface area monthly for a total of three or six doses in nine patients with PG. Seven patients had complete remission, one experienced failure, one had partial remission, and three had relapses after three and twelve months.^58^ However, rare but severe side effects, such as infertility, hemorrhagic cystitis, and secondary tumors, might limit its use.^3^

Dapsone and sulfasalazine were reported only in two patients with PG in Chile.^54^ However, recurrence occurred on multiple occasions and each patient was eventually transitioned to systemic steroids and cyclosporine.

For steroid-resistant PG, biological agents are now increasingly used in LA. TNF-α inhibitors are preferred due to availability and long-term safety data, but the high cost of these medications is still a limitation in this region.

## Conclusion

Based on the current studies, PG in LA is still an under-reported disease and there is a lack of robust studies. The most frequent form of PG is the ulcerative subtype, which is most commonly associated with IBD. An accurate diagnosis in LA is even more challenging, as the prevalence of cutaneous infections that mimic all forms of PG is higher compared to developed countries. Being aware of the epidemiological variations of infectious diseases might provide clinical clues in the diagnosis of PG-like lesions, not only in people from these areas but also in immigrants and travelers. Treatment of PG is primarily with systemic corticosteroids; however, some positive outcomes have been reported with cyclosporine and methotrexate. In LA, biological therapy data is scarce and apparently reserved for severe forms of PG non-responsive to steroids due to high cost and limited access.

## Financial support

None declared.

## Author's contributions

Milton José Max Rodríguez-Zúñiga: Approval of the final version of the manuscript.

Michael Heath: Approval of the final version of the manuscript.

João Renato Vianna Gontijo: Approval of the final version of the manuscript; composition of the manuscript.

Alex G. Ortega-Loayza: Approval of the final version of the manuscript; composition of the manuscript.

## Conflicts of interest

None declared.
